# Leveraging AI-driven predictors of enzyme pH optima to unravel microbial adaptation to environmental pH

**DOI:** 10.3389/fmicb.2026.1788098

**Published:** 2026-04-28

**Authors:** Peng Zhou, Zu-Xin Zhang, Xiaoyong Pan, Xinfeng Dai, Qiaoli Chen

**Affiliations:** 1 State Key Laboratory of Submarine Geoscience, Second Institute of Oceanography, Ministry of Natural Resources, Hangzhou, China; 2 Key Laboratory of Marine Ecosystem Dynamics, Ministry of Natural Resources and Second Institute of Oceanography, Ministry of Natural Resources, Hangzhou, China; 3 Institute of Image Processing and Pattern Recognition, Shanghai Jiao Tong University, and Key Laboratory of System Control and Information Processing, Ministry of Education of China, Shanghai, China; 4 Ocean College, Zhejiang University, Zhoushan, China

**Keywords:** artificial intelligence, enzyme pH optimum, eukaryote, pH adaptation, prokaryote

## Introduction

It is well-known that pH (potential of hydrogen) influences enzyme catalytic activity (Schomburg and Salzmann, 1991; Nelson et al., 2021). The pH optimum (pH_opt_), at which an enzyme displays maximal catalytic activity, is therefore critical for enzyme design and applications (Zhang et al., 2025). To identify suitable enzymes for target pH environments or optimize enzymatic performance in biotechnology, enzyme engineering requires efficient characterization of kinetic properties across large numbers of amino acid sequences. However, experimental determination of pH_opt_ for numerous sequences is time-consuming, labor-intensive, and costly. To address these limitations, computational approaches based on machine learning have been developed for rapid prediction of enzyme pH_opt_, supporting applications in protein engineering.

Recent advances, exemplified by EpHod (enzyme pH optimum prediction with deep learning) (Gado et al., 2025), enable prediction of the enzyme pH_opt_ directly from protein sequences. The EpHod model leverages embeddings from the protein language model (PLM) ESM-1v and achieves a root mean squared error (RMSE) of 1.25 pH units on the held-out test data (Gado et al., 2025). To further improve predictive performance, an increasing number of AI-driven tools have been developed. For instance, (Zhang et al. 2025) introduced the model V_ENUS_-DREAM, which employs the PLM ESM-2 and reduces the RMSE to 0.809. These AI-powered tools are revolutionizing enzyme discovery and design by enabling high-throughput prediction of pH_opt_.

Similarly, studies of microbial adaptation to environmental pH frequently require knowledge of enzyme pH_opt_. This information can be used to investigate the underlying adaptive mechanisms. However, experimental determination of pH_opt_ for large-scale enzyme sequences remains impractical due to high costs and low throughput. Fortunately, the high-throughput predictive capacity of these AI-driven tools offers a powerful alternative for obtaining enzyme pH_opt_ values, which can facilitate investigations into the mechanisms by which microorganisms adapt to environmental pH. The potential applications are illustrated with examples below.

## Potentials of AI-powered enzyme pH_*opt*_ predictors in elucidating pH adaptation mechanisms

To illustrate the value of AI-powered enzyme pH_opt_ prediction in understanding microbial pH adaptation, we selected three enzymes whose pH_opt_ values were accurately predicted by EpHod. The first example is the acid protease Thermopsin (UniProt: P17118), purified from the culture supernatant of *Sulfolobus acidocaldarius* strain DSM 639 (Lin and Tang, 1990). *S. acidocaldarius* strain DSM 639 was isolated from Locomotive Spring in Yellowstone National Park, USA, where the spring has a pH of 2.4, and exhibits optimal growth at pH 2-3 (Brock et al., 1972). Thermopsin acts as a secreted enzyme that digests extracellular proteins to provide nutrients for the cell (Rawlings, 2013; Tang and Lin, 2013). A logically relevant question is whether secreted Thermopsin can function effectively under such acidic conditions and thereby contribute to the adaptation of strain DSM 639 to its acidic habitat. Notably, the pH_opt_ of Thermopsin predicted by EpHod is consistent with experimental observations, which showed that this enzyme displays maximal proteolytic activity at pH 2 (Lin and Tang, 1990)—a value that agrees well with the in situ environmental pH. This consistency between the AI-predicted and experimentally determined pH_opt_ supports that EpHod can help reveal microbial niche adaptation, which is further reinforced by the second example presented below.

The second example is the acid-resistant membrane-bound amylopullulanase Apu (UniProt: Q4J9M2), which is also produced by *S. acidocaldarius* strain DSM 639. The purified Apu catalyzes the hydrolysis of starch, amylopectin, and pullulan into small oligomers for cellular uptake (Choi and Cha, 2015). EpHod again yielded an accurate pH_opt_ prediction for Apu, which matched the experimentally determined pH_opt_ value of approximately 3.0 for its amylolytic activity toward starch. The low pH_opt_ values of both Thermopsin and Apu ensure their catalytic activity under the acidic growth conditions of strain DSM 639, conferring a distinct physiological advantage for strain DSM 639 thriving in its native habitat.

The third example is an extracellular pectate lyase PelA (UniProt: D0VP31) from the alkaliphilic bacterium *Bacillus* sp. N16-5. The strain N16-5 was isolated from sediment of Wudunur Soda Lake in Inner Mongolia, China, and grows well within a pH range of 8.5–11.5 (Ma et al., 1991). The purified PelA from the culture broth of *Bacillus* sp. N16-5 efficiently depolymerizes polygalacturonate and pectin with digalacturonate and trigalacturonate as the main products (Li, 2010), and transcriptomic analysis of *Bacillus* sp. N16-5 grown on pectin indicated that these products could be subsequently imported into cells for metabolism, thereby supporting carbon acquisition (Song et al., 2013). EpHod also accurately predicted the pH_opt_ of PelA, which was in good agreement with the experimentally determined value of 11.5 (measured at 50 °C using polygalacturonic acid as the substrate) (Li, 2010). The high pH_opt_ of PelA enables its robust function in alkaline environment, thereby promoting the survival and adaptation of *Bacillus* sp. N16-5 in its ecological niche.

Collectively, these three examples suggest that AI-powered enzyme pH_opt_ prediction could advance our understanding and inference of microbial adaptive strategies to environmental pH conditions. Additional enzymes with reliably predicted pH_opt_ values by EpHod are available in the full dataset (Zenodo: doi: doi: 10.5281/zenodo.14252615; Gado et al., 2023). Leveraging the utility of advanced AI-powered pH_opt_ prediction tools like EpHod not only helps researchers to further deepen mechanistic insights into microbial adaptation to environmental pH, but also provides users with valuable guidance in a rapid and cost-effective manner for experimental design, such as the selection of candidate enzymes for follow-up research. These practical applications raise higher requirements for model performance and highlight the need for further model improvement, given the presence of discrepancies between predicted and experimentally determined pH_opt_ values.

## Advancements and prospects of AI-driven enzyme pH_opt_ prediction

Continuous efforts have been devoted to improving the performance of AI-driven enzyme pH_opt_ prediction models. For instance, since enzymes usually function over a range of pH values rather than at a fixed point, Wang et al. developed DeepPH, an interval-aware prediction framework that better reflects the inherent nature of pH_opt_ as a range (Wang et al., 2025). DeepPH uses a spatial radius-based structural encoding scheme and a multimodal attention pooling for fusing sequence and structure information. Evaluations on test datasets demonstrated that DeepPH outperforms both EpHod and V_ENUS_-DREAM in both average-based and interval-aware pH prediction tasks (Wang et al., 2025).

In addition to the continuous improvement of model architectures and algorithms, the PLMs employed should also be advanced. For example, ESM-1v and ESM-2, used in enzyme pH_opt_ prediction, do not take post-translational modifications (PTMs) into account. In reality, enzymes frequently undergo various PTMs to fulfill their essential biological functions. These PTMs encompass a wide variety of chemical modifications, including acetylation, phosphorylation, glycosylation, methylation, and ubiquitylation (Macek et al., 2019), and are ubiquitous within cells (Beltrao et al., 2012). Enzyme pH-activity profiles can be significantly modulated by chemical modifications (Xue et al., 2010; Li, 2014; Giri et al., 2021). Therefore, to improve the accuracy of pHopt prediction, it is essential to equip predictors with the ability to incorporate PTMs. A promising strategy for integrating PTM information into embedding processes is to use PTM-aware PLMs. A critical step in this direction is the precise mapping of PTM annotations to corresponding protein sequences. Recently, (Peng et al. 2025) proposed PTM-Mamba, a PLM that integrates PTM tokens using bidirectional Mamba blocks fused with ESM-2 embeddings via a gating mechanism. PTM-Mamba has strong potential as a foundational PLM for various downstream PTM-aware tasks, with enzyme pH_opt_ prediction being a prominent application.

It should be noted that in addition to pH, other environmental factors, such as temperature or ionic strength, can also influence enzyme activity (Ishigami and Morita, 1977; Ivanov et al., 2026); thus, experimental validation remains indispensable. Direct experimental determination of pH optima provides solid and reliable results that cannot be replaced. On the other hand, as more experimentally measured pH_opt_ values accumulate and are incorporated into training datasets, the performance of such AI predictors will continue to improve.

## Discussion

Microorganisms have evolved diverse strategies to adapt to environmental pH. As essential biological catalysts, enzymes play a pivotal role in biochemical reactions. Knowledge of enzyme pH_opt_ is a key to understanding the dynamic interplay between enzymes, pH, and biological adaptation. Investigating pH_opt_ is therefore highly valuable for uncovering the mechanisms of microbial adaptation. However, experimental determination of pH_opt_ for large-scale enzyme sequences (e.g., those annotated from genomes or metagenomes) is highly time-consuming, labor-intensive, and expensive. Using these AI-powered prediction tools enables time-efficient, cost-effective, and high-throughput prediction of pH_opt_ values. Here we discuss the application potential of advanced AI-driven pH_opt_ predictors in studying the adaptive strategies employed by microorganisms to cope with environmental pH. High-throughput predictions facilitate researchers in inferring the functional roles of enzymes in pH adaptation and conducting hypothesis-driven studies to explore the adaptive strategies of microorganisms. For example, from a pH_opt_ perspective, the significance of isoenzymes that catalyze identical reactions but exhibit distinct pH_opt_ values can be systematically evaluated.

Moreover, such high-throughput predictions allow researchers to investigate the competitive advantages of different organisms under specific environmental pH conditions and their implications for species succession. For instance, elevated pH differentially affects the growth and survival of various marine phytoplankton species (Hansen, 2002; Zepernick et al., 2021). Based on pH_opt_ prediction, the molecular mechanisms underlying the competitive advantages of alkalitolerant bloom-forming algal species under elevated water pH can be analyzed, improving the mechanistic understanding of algal bloom outbreaks. Furthermore, ocean acidification poses a severe threat to marine ecosystems, especially to calcifying organisms (Orr et al., 2005). Coral reefs, among the most vulnerable marine ecosystems, are being severely impacted (Hoegh-Guldberg et al., 2007). Using AI-predicted enzyme pH_opt_ values, we can analyze the match between enzyme pH_opt_ and fluctuating seawater pH, thereby inferring species adaptability and the adaptive succession of biological communities under ocean acidification.

In summary, enzyme pH_opt_ prediction tools enable a deeper understanding of the mechanisms by which organisms cope with environmental pH. As the field of AI-driven pH_opt_ prediction continues to advance, novel insights will be gained to facilitate the elucidation of pH adaptation mechanisms, potentially leading to important scientific breakthroughs.
